# Cellular plasticity and transcriptional reprogramming in plant-nematode interactions: insights into feeding site formation and plant defense

**DOI:** 10.1007/s44297-025-00056-1

**Published:** 2025-07-21

**Authors:** Abdulmujib Gboyega Yusuf, Tesleem Taye Bello

**Affiliations:** 1 Plant Protection Department, College of Food and Agricultural Sciences, King Saud University, P.O. Box 2460, Riyadh, 11451 Saudi Arabia; 2 Department of Agricultural Science Education, Federal College of Education, PMB 2096, Ogun State Abeokuta, Nigeria

**Keywords:** Plant-nematode interactions, Transcription factors, Cellular reprogramming, Plant immunity modulation, Nematode feeding site, Root-knot nematode

## Abstract

Plant-parasitic nematodes, especially sedentary endoparasites, threaten global agriculture by inducing cellular plasticity in host plants to form specialized feeding structures. Sedentary nematodes such as root-knot and cyst nematodes establish feeding sites, including giant cells and syncytia, to extract nutrients from the host. Feeding site formation involves complex biological processes, including cell cycle activation, metabolic reprogramming, cytoskeleton rearrangement, and hormonal signaling. This review explores the underlying molecular mechanism driving plant cellular plasticity, focusing on the role of the transcription factors that regulate gene expression during organogenesis, peculiar to giant cells and syncytia, essential for the nematode's sustenance during the sedentary life stage. Key transcription factors, including members of the MYB, WRKY, ARF, ERF, and LBD families, are modulated by nematode effectors during compatible interactions to reprogram plant gene expression to facilitate the development of the nematode feeding site. Despite the roles of transcription factors in establishing feeding sites, they present other roles in regulating plant defense responses, thereby balancing growth reprogramming with the activation of plant immune signaling pathways. The review also highlights the allowance limit of plant physiological processes during cellular reprogramming and defense response, providing insights into how certain plants can resist nematode infection. Furthermore, emerging biotechnological strategies, including molecular breeding and gene editing, are discussed as potential approaches to disrupt nematode-induced reprogramming, highlighting novel avenues for enhancing crop resistance. Understanding the molecular mechanism and physiological dynamics between cellular plasticity and transcriptional regulation in plant-nematode interactions is essential for developing sustainable solutions to mitigate the impact of plant-parasitic nematodes on agricultural production.

## Introduction

Plant parasitic nematodes (PPNs) are among the most devastating agricultural pests and are responsible for significant losses in agricultural production worldwide. They are microscopic wormlike organisms that infect plant roots mainly through the action of their protractible stylets in piercing plant tissue, depositing molecules known as effectors into host cells, and extracting nutrients. Generally, PPNs are categorized as endoparasites and ectoparasites depending on their active location within or on the plant's surface. However, the endoparasitic nematodes are agronomically more important because they comprise the obligate group capable of inducing physiological and morphological changes in host cells to create specialized feeding structures [1]. Two of the most well-characterized feeding sites are giant cells, formed by root-knot nematodes (RKN; *Meloidogyne* spp.), and syncytia, induced by the cyst nematodes (CN; *Globodera* and *Heterodera* spp.) [2–4]. These structures are emergency sinks that serve as the nematode's nutrient source through the parasitic life cycle, making them necessary for its development and reproduction [5]. Root-knot nematode giant cells present a remarkable ontogeny, forming through a series of mitoses lacking cytokinesis and repeated endoduplication [6, 7]. Notably, the deposition of the secondary cell walls, which form xylem ingrowth essential for solute transport, is accompanied by nuclear and cellular hypertrophy. This process is surrounded by actively dividing root cells, ultimately leading to gall formation [8]. On the other hand, CN manipulates host physiology into the formation of the syncytium, observable as a multinucleate fusion of host cells that have had their cell walls dissolved partially [9, 10]. The amalgamated cells can be up to several hundred, including the initial cell injected and other adjacent cells.

Feeding site formation exemplifies a remarkable form of cellular plasticity in plants, where infected plants undergo extensive reprogramming orchestrated by the RKN intervention. Generally, cellular plasticity is the ability of a cell to modify its morphology and function in response to external stimuli, allowing the cell to transition between different phenotypic states [11, 12]. Specifically, in the context of plant-nematode interaction, this plasticity is manipulated by nematodes to reconfigure plant cells into feeding sites, making them more suitable for parasitism. During this process, the normal developmental pathways of plant cells are subverted, leading to alterations in the cell cycle, cell wall architecture, and metabolic activity [3, 8].

This intricate reprogramming underlies a cross-kingdom communication between nematodes and their host plant, where compatibility plays a keen role in determining the nematode's success. For instance, in incompatible interaction, i.e., when resistance is met, more males emerge from the developing juveniles, which is a detriment for the perpetuation of the nematode species [13, 14]. If compatible, host transcriptional processes are modulated by nematode effectors to activate, regulate, or dysregulate the transcriptional program such that the host cellular machinery is reprogrammed to form feeding sites for the nematode [15]. In any of the roles, the nematode effectors have to interact with items in the host, particularly those associated with cellular processes of plant defense and development. Therefore, host cellular alteration is important for the nematode, and there is a paucity of information on the mechanism of the host-nematode interaction that leads to cellular plasticity.

A critical aspect of this nematode-induced reprogramming is the role of transcription factors (TFs), which serve as master regulators of gene expression upon their interaction with RKN effectors [6, 16]. Despite its seemingly simple role, TFs control a wide array of cellular processes by binding to specific DNA sequences and activating or repressing the transcription of target genes, leading to a change in innate plant response [17, 18]. Interestingly, in nematode-infected plants, several TFs are either hijacked by the nematode or activated as part of the plant's response to infection. These factors orchestrate the cellular changes required for feeding site development, including cell proliferation, differentiation, and nutrient transport by RKN-induced transcriptional activation of genes that are key players in cell cycle regulation [2, 19]. Key transcription factors such as those from the WRKY, ethylene response factor (ERF), lateral organ boundaries domain (LBD), and MYB families have been implicated in these processes [20–22].

In parallel with the induction of cellular plasticity, infected plants can also mount immune responses to limit nematode invasion. These defense mechanisms often involve the activation of hormonal signaling pathways (e.g., salicylic acid (SA), jasmonic acid (JA), and ethylene (ET), which play critical roles in regulating plant immune responses [23]. Although acting downstream, TFs are known to activate several plant hormones in planta, transcriptome analysis has proven that defense-associated genes are often repressed in locations of infection, such as giant cells and galls [21, 24]. Needless to say, RKNs have evolved sophisticated strategies to suppress these defense responses by repressing the TFs, often through the secretion of effector proteins that interfere with the plant’s immune machinery. For instance, the WRKY45 gene expressed during tomato plant early infection by *M. javanica* is observed to be associated with a decrease in SA- and JA-defense marker genes [18]. This creates a delicate balance between the plant's attempts to defend itself and the nematode's effort to manipulate host cellular machinery.

Despite significant advances in understanding nematode-induced cellular reprogramming, there is much to uncover about the molecular mechanisms underlying these processes. Specifically, the exact roles of transcription factors in modulating the plant's developmental pathways during nematode infection and how these factors integrate with the plant's defense responses should be areas of active research. Therefore, this review aims to explore the cellular plasticity and transcriptional reprogramming that occur during plant-nematode interaction, focusing on the key TFs involved in forming nematode-feeding sites. By examining how nematodes manipulate plant transcriptional networks to induce feeding site formation and how plants attempt to balance these changes with defense, the review seeks to provide new insights into the molecular interplay between plant hosts and their nematode parasites. Additionally, it will discuss how advances in our understanding of these processes could inform future strategies for developing nematode-resistant crops through molecular breeding and genetic engineering.

### Molecular interplay between plant defense and nematode-induced reprogramming

Generally, plants possess innate resistance to nematode invasion that routes various defensive approaches such as pattern-triggered immunity (PTI) and effector-triggered immunity (ETI) [25]. Primarily, plant cells can recognize pathogen-associated molecular patterns (PAMPS), which subsequently activate the PTI [26]. Consequently, defense responses, including the production of reactive oxygen species (ROS) and cell wall reinforcement, are initiated [27, 28]. Nonetheless, PTI is liable to succumb to nematode invasion when nematodes secrete their effector proteins beyond the first line of defense [29, 30]. However, the plants are equipped with resistance genes (R) that mediate the activation of a deeper defense reaction, the ETI, to include localized cell death [25]. Essentially, they must suppress the plant defense pathways for the nematode to establish successful feeding sites. Remarkably, with the aid of their effectors, nematodes can interfere with plant hormone signaling pathways, particularly salicylic acid (SA), jasmonic acid (JA), and ethylene (ET), which are critical to defense regulation [31]. Inevitably, the plant's immune responses are weakened, and the nematode can affect cellular reprogramming essential for developing the nematode's feeding sites.

Despite the seemingly simple appearance of the feeding site establishment, there is significant cross-talk between defense signaling and the cellular changes induced. For this process to occur, hormonal regulation is vital, with auxin and cytokinin being relevant to feeding site formation, while the SA, JA, and ET-dependent defenses are suppressed [32, 33]. Additionally, transcription factors such as WRKY, NAC, MYB, and AP2/ERF mediate both defense responses and the reprogramming of plant cells [19], where signals from immune pathways are integrated to balance plant growth and defense. All taken together, effective suppression of plant defense will enable susceptible plants to allow feeding site development, and resistant plants can withstand the nematode invasion through rapid immune activation. Thus, the complex molecular interplay is responsible for the outcome of plant nematode interactions, driving either compatibility or resistance.

### Cellular plasticity in response to nematode infection

#### Nematode-Induced Feeding Sites: Giant Cells and Syncytia

Sedentary endoparasitic nematodes induce drastic cellular reprogramming in host root tissue to establish specialized feeding sites (Giant cells and syncytia). These feeding sites are formed through various manipulations of plant developmental processes that exploit the inherent plasticity of plant cells. A hallmark of this interaction is the ability of differentiated plant cells to dedifferentiate, re-enter the cell cycle, and transdifferentiate into specialized transfer cells [34]. This reprogramming underlines the formation of enlarged, multinucleated, and transcriptionally active feeding cells supporting long-term nematode parasitism [35]. In a specialized manner, the nematodes, with the aid of their stylet, perforate the plant cell to withdraw the required material for the nematode's survival and to deposit several molecules and specific proteins otherwise known as effectors. In particular, the secreted protein mixture contains cellulose, hemicellulose, and pectic-degrading enzymes, which specialize in accelerating cell digestion and expansion [36–38]. The effectors are secreted from the dorsal and sub-ventral esophageal gland cells to which the stylet is connected to serve as an outlet for the secreted molecule [32, 39]. The esophageal gland is a single cell with an elongated cytoplasmic extension that collapses into an ampulla [32]. Notably, effectors that manipulate plant physiological responses are secreted from the dorsal glands [39].

Infection begins when the infective second-stage juveniles invade the root tip and migrate toward the vascular cylinder. Root-knot nematodes typically move intercellularly and induce redifferentiation of several parenchyma cells into hypertrophied, multinucleated GCs via nuclear division without cytokinesis. This has been confirmed through analysis of mitotic markers, nuclear content, and cell wall stubs [2, 7]. In contrast, CN juveniles penetrate the root and migrate intracellularly to the vascular cylinder, where they induce local dissolution of cell walls, alongside the fusion of protoplasts of adjacent cells to initiate syncytia formation [30, 40]. The feeding sites are metabolically hyperactive locations that support the nematode's nutrient demands to complete its development. They display distinct morphological and physiological features such as increased cytoplasmic density, altered vacuole structure, and reorganization of cytoskeletal elements [41]. During early GC formation, the central vacuole fragments into smaller vacuoles, resulting in a dense cytoplasm that facilitates enhanced metabolic activity and efficient nutrient transfer [7, 42]. At the onset of giant cell development, observable changes, including cell wall thickening, occur in the cell wall proximity of phloem and xylem elements, which may be necessary for the transfer of water and other solutes [43, 44]. Also, there is the proliferation of cell wall ingrowth during early RKN development, which later degrades towards the completion of the nematode life cycle [44]. Essentially, the cell wall ingrowth, a hallmark of GC, increases the surface area of the plasma membrane, and it is vital to the exchange of nutrients between the feeding cells.

Similarly, in the syncytium of CN, there is a significant alteration to the plant cell wall, including cell wall dissolution during nematode development. While nematode-derived cell wall-degrading enzymes are strongly implicated in host tissue penetration and migration, their direct role in feeding site establishment remains to be fully elucidated [45, 46]. All together, these host-nematode dialogs yield a continuous relationship between the two, which benefits from the plasticity of the plant cells. Thus, establishing and maintaining feeding sites rely on extensive reprogramming of host developmental pathways, including those regulating cell size and shape, cytoskeletal organization, and vascular connectivity [47].

Phenotypically, cells increase dramatically in size and occupy a large portion of the root cortex. Usually, syncytium encompasses 200 cells and above at its maximum size [48]. Cabrera et al. [49] reported that GC volume can expand and increase to about 60-fold between 3 to 40 days after infection. A phenomenon that occurs due to cell wall loosening and cytoskeleton rearrangements. More so, the increment in cell volume allows for an extensive cytoplasmic activity required for nutrient absorption by the nematodes. The dynamism exhibited by the cytoskeleton lies in its continuous rearrangement, which has been observed to include three components: actin microfilaments, microtubules, and intermediate filaments [50, 51]. However, the rearrangement could be a result of stimuli other than nematode invasion, such as wounding [52], hormonal treatment [53], and symbiosis [54], all with a link to plant responsive mechanisms. A composite phenomenon that highlights the admirable nature of cell plasticity. Notably, an intricate connection exists between the feeding sites and the plant's vascular tissue that supports water, mineral, and organic compound movement [47]. Vascular tissues are popular for their roles in signal transmission and communication between organs temporally and spatially, where they mediate nutrient distribution throughout the plant [55]. Altogether, GC and syncytia represent highly specialized cellular niches, shaped by coordinated changes in host gene expression, cytoskeletal architecture, and vascular integration. These intricate systems show the remarkable plasticity of plant cells and highlight the complexity of nematode-host interaction.

#### Signaling pathways governing plant cell plasticity

It is expected that the dramatic morphological changes expressed in feeding sites should feature significant signaling pathways leading to the substantial plasticity induced by both RKN and CN and the plant response. Therefore, understanding the pathways during infection is essential to understanding plant-nematode interaction. Nematodes interact with plants in a sophisticated manner to overcome their defenses and modulate their metabolism and physiology [56]. For the plant cells to submit to the strange post-embryogenic organogenesis occurring in the feeding site, nematodes manipulate the various plant cells'regulatory mechanisms, including cell cycle, physiological programs, and stimulus responses [19]. The reprogramming of plant cells in response to nematode manipulation is conducted by multiple signaling pathways regulated by plant hormones. A significant part of this reprogramming is regulated by plant hormone signaling. However, hormonal signaling operates with other intracellular signaling mechanisms such as reactive oxygen species (ROS) and Ca^2+^ signaling, which have emerged as critical components in the regulation of plant cellular plasticity.

Plant hormones play crucial roles in regulating cell plasticity during nematode infection, with auxin, ethylene, gibberellin, and cytokinin emerging as key regulators in the formation of nematode-feeding sites [32, 39]. These phytohormones are essential in activating diverse aspects of plant cellular processes, including cell cycle re-entry, division, differentiation, and expansion, which are all critical for establishing the feeding structures formed during nematode infection [57, 58]. A pivotal factor in this activation is the local accumulation of auxin, a master regulator that plays a central role in transcriptional reprogramming and organogenesis [59]. The auxin receptor, transport inhibitor response 1 (TIR1), associated with endogenous auxin accumulation, is correlated with increased nematode susceptibility [60, 61]. Similarly, the expression of *AUX*_*1*_ auxin importer is induced in nematode-infected cells during early NFS establishment, facilitating auxin transfer into nematode-selected cells [62, 63]. This process triggers the activation of cell wall-modifying proteins, promoting pectin polymerization and cell wall loosening, all to enable the required cell expansion for NFS development [64, 65].

Cytokinin coordinates synergistically with auxin to regulate cell division, differentiation, and cell cycle activation [35, 66], particularly at the G1/S-phase transition [67]. In a way, nematodes can influence cell division by triggering cytokinin synthesis and modulating downstream signaling events that activate the division. Supporting this, the silencing of isopentenyltransferase (cytokinin biosynthesis) in *H*. *schachtii*, suppressed NFS expansion, and plant mutants deficient in cytokinin signaling demonstrated significant resistance to CN infection [32, 68]. Additionally, mutants lacking cytokinin receptor genes *AHK3/4* show reduced cell cycle reactivation and susceptibility to CN infection [68]. However, using a cytokinin-sensitive marker construct, *ARR5* expression is notably absent in mature giant cells, implying cytokinin’s primary role in the early stages of NFS development [69]. This signifies that cytokinin, among other phytohormones, is important to cell cycle activation. The interplay between auxin and cytokinin signaling is critical not only in lateral root development but also in the coordinated expansion and maintenance of NFS.

Gibberellin (Gibberellic acid; GA) is also implicated in nematode parasitism, particularly through the upregulation of GA12 in nematode-infected tissues [70]. Ethylene (ET) is also a key hormone in nematode-plant interactions, playing dual and sometimes contradictory roles. It has been implicated in attracting beet cyst nematode (BCN) and RKN to plants'roots [71, 72]. Overproducing ET in *Arabidopsis* increased susceptibility to CN infection, resulting in syncytia featuring extensive cell wall expansion [69]. In contrast, early signs of ET signaling were found in Arabidopsis roots after nematode infection, suggesting that ET signaling is responsible for reduced attractiveness to SCN [73]. Also, through interaction with the ethylene receptor ETR1, ET can inhibit cytokinin-mediated processes, reducing *H*. *schachti*i infection [74]. These findings highlight the ET complex's role as a promoter of cell wall modifications and a potential elicitor of defense mechanisms. Resolving this ambiguity requires further investigation into ET precise regulatory role.

**Fig. 1:**
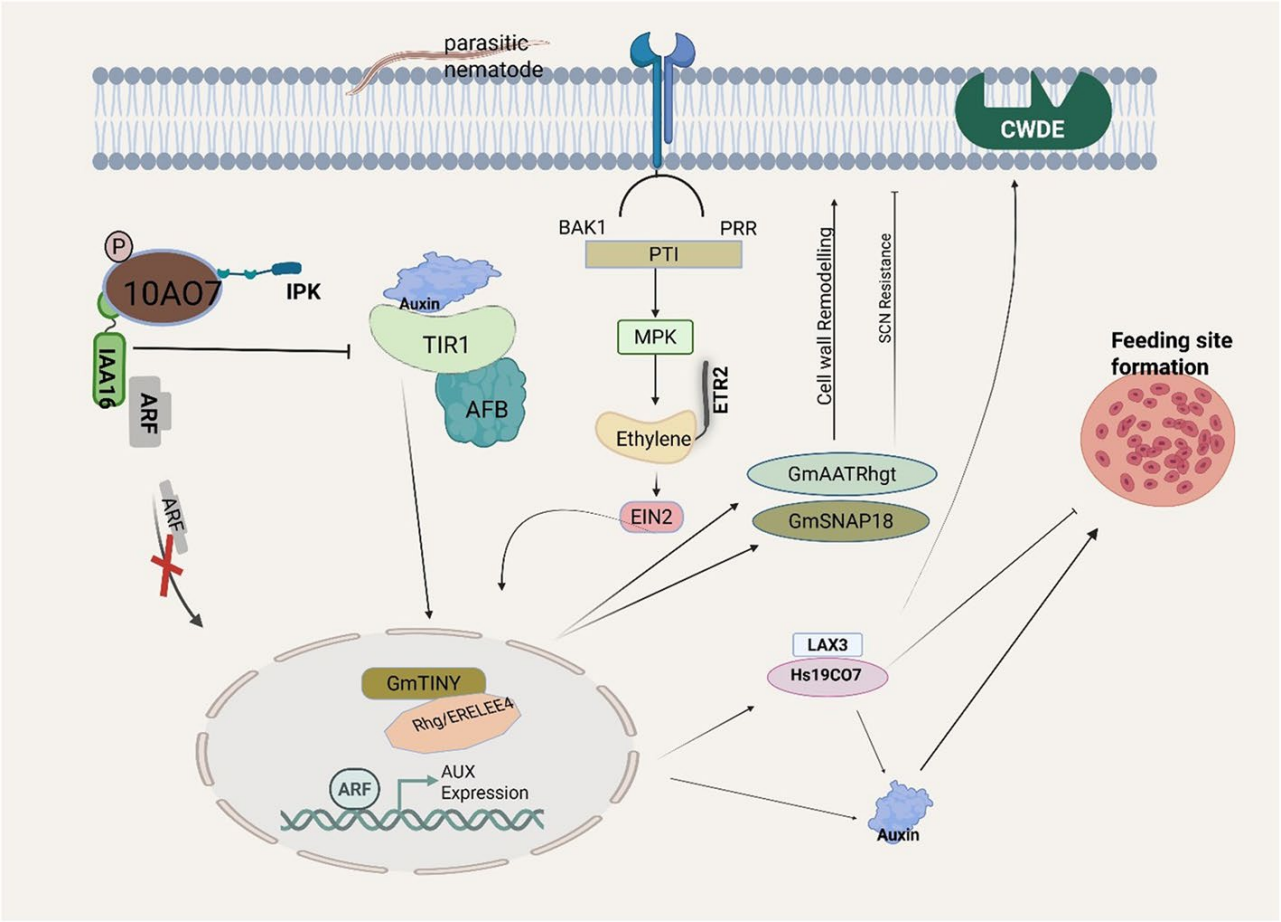
Effector-Mediated Transcriptional reprogramming and Hormonal Signaling During Nematode Parasitism. During nematode infection, auxin transport is triggered to facilitate feeding site formation, requiring significant gene expression reprogramming. Auxin, a key player in this process, is modulated through various mechanisms involving nematode effectors. The effector 10AO7 interacts with the auxin response suppressor IAA16, destabilizing its suppressive effect on ARFs and enhancing auxin signaling. This modulation is further supported by phosphorylation from IPK kinase, which activates 10AO7. Similarly, the effector Hs19C07 targets auxin influx carrier LAX3, enhancing auxin transport into infected cells. LAX3 also has a role in regulating cell wall-degrading enzymes. Ethylene signaling also has a role in host response activation. Nematode infection triggers ethylene synthesis through the ETR2/EIN3 pathway. The AP2/ERF TF GmTINY, whose expression is induced by the SCN infection, binds to ERELEE4 elements within the Rhg1 locus, to activate the synthesis of GmAATRhgt and GmSNAP18. Interestingly, auxin signaling can also activate the expression of GmTINY, signifying hormonal crosstalk. These coordinated actions highlight how nematodes manipulate plant hormonal networks and transcriptional mechanisms to promote parasitism, and plants'responses via regulated defense pathways. Created in https://BioRender.com

Importantly, these hormonal pathways do not function in isolation. Rather, crosstalk between hormonal networks and nematode effectors reprograms the cellular environment to favor nematode development. Effectors play critical roles in modulating host cellular plasticity, ensuring the formation and functionalization of feeding site structures. The establishment of these sites requires precise hormonal homeostasis, which nematodes achieve by targeting key components of phytohormone signaling networks [75]. Early syncytium development has been attributed to changes in auxin dynamics, as nematode effectors are known to influence auxin synthesis, transport, and signaling (Fig. 1). For instance, *H. schachtii* effector Hs19C07 interacts with the auxin transporter LAX3 (an auxin influx protein (AUXI)), to trigger auxin influx and upregulates cell wall-degrading enzymes (CWDE), facilitating cell wall remodeling during early syncytium formation [35, 76].

Further evidence of auxin pathway manipulation from *H*. *schachtii,* where the effector 10A07 interacts with an auxin suppressor factor IAA16 and a plant protein kinase IPK. This interaction enhances parasitism by facilitating effector trafficking to the nucleus, where it interferes with auxin-responsive transcription factors [77]. The involvement of signaling mechanisms, such as ROS and Ca^2+^, in the manipulation of plant cells by sedentary nematodes is a complex interplay that facilitates nematode parasitism [78]. ROS are produced in response to biotic stress to activate programmed cell death pathways, which the nematode suppresses to ensure feeding site establishment [79].

Calcium signaling is a primary response to biotic stress, acting in transmitting signals that activate defense genes [80]. The modulation of Ca^2+^ transients by nematodes can lead to changes in plant cell physiology to promote the development of GC and syncytia. For example, the conductance of Ca^2+^ across cell membranes has been linked to auxin-induced ROS production in roots [81]. Together, these findings highlight the complex hormonal cross-regulation and effector interplay underlying nematode-induced cellular plasticity. Understanding these complicated interactions will provide valuable insights into host manipulation strategies employed by nematodes.

#### Suppression of plant immunity

Nematode also employs effectors to suppress host innate immunity, particularly by scavenging reactive oxygen species (ROS) generated during the oxidative burst that follows pattern-triggered immunity (PTI). For example, in rice, the effector MgMO237 from *M*. *graminicola* suppresses PTI by interacting with endogenous defense-related proteins such as OsGSC (1,3 beta-glucan synthase), OsCRRSP55 (cysteine-rich repeat secretory protein), and OsBetvl (pathogenesis-related Betvl family protein) [82]. Similarly, the effector MgMO289 interacts with the rice heavy metal-associated protein OsHPPo4 to manipulate the host ROS system to suppress rice immunity [83].

There is also the *M*. *javanica* effector MjTTL5 that interacts with the thioredoxin reductase catalytic subunit (AtFTRc) to scavenge ROS during the oxidative burst event that follows PTI, thereby suppressing the plant defense responses [84]. A similar pathogenic pathway is observed in CN. Sugar beet cyst nematode, where the effector 10Ao6 interacts with a specific spermidine synthase2 (SPDS2), a polyamine biosynthesis enzyme, to enhance the cellular antioxidant capacity at the infection site [85]. In *G*. *rostochiensis*, the effector ubiquitin carboxyl extension protein (GrUBCEP12) of derivative GrCEP12 suppresses flg22-induced PTI [86]. Another effector, Gr29D09 of the same nematode, also targets the hexokinase 1 protein (StHXK1) via a similar pathway to suppress plant immunity [87]. However, the specific host target of the nematode effector, GrUBCEP12, remains unidentified. Further studies are necessary to uncover the precise mechanisms by which GrUBCEP12 modulates plant immunity and to identify its specific targets.

Another notable effector, Mg16820, exemplifies the dual functional localization of nematode effectors. As an apoplast-transported effector, Mg16820 acts as an immune suppressor by suppressing the induction of flg22-induced ROS production while targeting a dehydration stress-inducible protein (DIP1) in the cytoplasm to modulate the host stress response [88]. This further demonstrates the sophisticated strategies nematodes use to suppress plant defense.

While significant progress has been made in understanding nematode effector and their interactions with plant hormonal and immune pathways, there are still some gaps to fill. For instance, the specific targets of certain effectors, such as GrUBCEP12, are yet to be identified. Further studies to identify the host target for GrUBCEP12 could aim to look into the Ubiquitin–proteasome system (UPS), which is a conserved host target for several pathogens'effectors. Notably, the *G*. *pallida* effector GpRbp1 is known to interact with a UPL3 homologue of the proteasome system [89]. This insight could be an outlook for identifying various nematode effector targets. Additionally, the dual role of effectors like the Mg16820 emphasizes the complexity of host-nematode interaction, which requires the spotlight of studies to decipher their intricate functions. Developing resistant crop varieties by disrupting nematode signaling pathways, especially those involving plant peptide mimicry, holds promise as a sustainable strategy.

### Transcriptional dynamics during feeding site formation

Recent advances in plant-pathogen interaction have revealed that RKN and CN secrete a repertoire of effector proteins that hijack critical processes governing plant cell physiology, morphogenesis, and immunity. These effectors facilitate the establishment of feeding sites and ensure successful parasitism. The ability to monitor effector localization, from their secretion in nematode glands to their transit in planta and eventual delivery to the apoplast, has been significantly improved by in situ hybridization, immunolocalization techniques, and transcriptomics [31, 90, 91]. Such advancements have validated candidate effector genes and provided insights into the transcriptional regulation of plant responses during nematode infection [92]. However, while earlier studies emphasized the role of nematode effectors in breaking down plant tissues to facilitate penetration, migration, and suppressing plant defenses [35, 45, 93], comparatively less is known about how these effectors induce organogenesis and reprogram plant cells to support feeding site development.

Interestingly, Recent evidence suggests that nematodes manipulate evolutionarily conserved host protein targets known as “hubs” to mediate transcriptional reprogramming. For instance, the COP9 signalosome, particularly the CSN5 subunit, targeted by multiple microbial pathogens, also interacts with peptide effectors from diverse fungal and bacterial pathogens, with its subunit 5 (CSN5) being a common target [94, 95]. Similarly, other conserved hubs such as the TEOSINTE BRANCHED1/CYCLOIDEA/PROLIFERATING CELL FACTOR (TCP) and JASMONATE-ZIM DOMAIN (JAZ) families also serve as effector targets. In *Arabidopsis*, the effector Mj2G02 from *M*. *javanica* dysregulates JAZ and other JA-responsive genes to suppress plant immune responses [83]. Another effector, Hs25A01 from *H*. *schachtii*, interacts with the host translation initiation factor (ELF) family, elF-2bs, to increase nematode infection [96].

These conserved molecular interfaces exploited by nematodes and other pathogens highlight the strategic advantage nematodes gain by exploiting shared host vulnerabilities. Therefore, understanding effector/target interactions is vital in developing durable host resistance. However, beyond the effector target, the host TFs are a key regulator of transcriptional reprogramming that modulates cellular transition for nematode NFS formation.

Transcriptomic analyses across infection stages, from initial nematode invasion to gall development, have revealed extensive transcriptional reprogramming in host roots [19, 97]. Transcription factors play a central role in this reprogramming by genes associated with cell proliferation, differentiation, organogenesis, and immune suppression. TF families such as the GRAS, NAC, and MYB have been implicated in nematode-induced developmental changes. Understanding their specific roles during nematode parasitism offers promising targets for disrupting parasitic success and improving resistance traits.

#### Role of transcription factors in reprogramming plant cells

Plant defense responses rely on sophisticated signaling networks and precise transcriptional control. Transcriptional factors (TFs) are essential in mounting rapid responses to offensive stimuli [98]. Typically, plant defense begins with pathogen-associated molecular pattern (PAMP)-triggered immunity (PTI), involving MAPK cascades, ROS production, and hormone biosynthesis [99, 100]. There is also a second layer of plant defense known as effector-triggered immunity (ETI), creating a hypersensitive response that leads to localized cell death [79, 101]. Nematodes can overcome these layers by targeting the plant’s transcriptional machinery. They induce changes in pre-mRNA splicing and gene expression patterns to suppress basal, including pre-mRNA splicing and transcriptional reprogramming [102]. For instance, RKN induces splicing reprogramming of approximately 2,898 genes, involving over 9,065 transcripts, during gall formation and parasitism, emphasizing the nematode’s ability to modulate host gene expression [103]. Numerous TF families, including MYB, WRKY, bZIP, bHLH, NAC, and ERF/AP2 regulate key processes during parasitism (Table 1). These regulators can be activated, repressed, phosphorylated, or degraded depending on pathogen influence [104], illustrating the vulnerability of transcriptional networks to effector-mediated manipulation.

**Table 1:** Key transcription factors (TFs) involved in nematode-induced transcriptional reprogramming associated with feeding site formation

TFs Regulated	Plant/nematode	pathway	Assay/analysis	Expression/Role	Mechanism of modulation	Ref
MYB3R1	*Arabidopsis/M. incognita*	Hormone signaling	Functional analysis	Upregulation/Transcriptional activator	Interacts with CDKs, leading to phosphorylation, thereby enhancing the activity required for gene expression	[20]
MYB34	*Arabidopsis/M. incognita*	PTI response	Gene expression analysis	Upregulation/glucosinolate biosynthesis	As part of the defense response, signals associated with the nematode infection modulate the expression of MYB34	[105]
MYB93	*Arabidopsis*	Auxin signaling	Transcriptome analysis	Upregulation/Regulates the expression of cell wall organization genes	Regulates the expression of the cell wall gene EXPA17	[106]
SIWRKY3	*Tomato/M. javanica*	Hormone signaling	Functional analysis	Upregulation/positive regulator of resistance through shikimate pathway activation	Accumulation of defense-related metabolites from the shikimate and oxylipin pathways during overexpression	[17]
SLWRKY72a/SLWRKY72b	*Tomato/M. incognita*	Mi-1 triggered ETI	Microarray/functional analysis	Upregulation/Transcriptional activator of immune responses	There is an interaction between SLWRKY72a and SLWRKY72b, translating to a functional dependency, which is possible through heterodimer formation and concurrent modulation of activity	[107]
OsWRKY34	Rice/*M. Graminicola*	Phytohormone signaling	Gene expression analysis/functional analysis	Upregulation/positive regulator of innate immunity	Upon infection, defense-related genes are expressed, triggering the modulation of the hormonal defense pathway	[108]
OsWRKY36	Rice/*M. Graminicola*	Phytohormone signaling	Gene expression analysis/functional analysis	Upregulation/positive regulator of innate immunity	Contribute to the defense mechanism by inducing oxidative stress response via upregulation of genes related to peroxidases and glutathione S-transferases	[108]
WRKY17	*Arabidopsis/M. incognita*	Glucosinolate/camalexin biosynthesis via PTI	Gene expression analysis	Downregulation	Regulates glucosinolate biosynthesis in a BAK1-dependent way	[18]
WRKY11	*Arabidopsis/M. incognita*	Glucosinolate/camalexin biosynthetic pathways	Gene expression analysis	Upregulation/positive regulator of basal defense	In a BAK1-dependent manner, WRKY17 is down-regulated as a positive regulator of genes involved in the biosynthesis of antimicrobial compounds in the syncytia	[105, 109]
SIWRKY16	Tomato/*M. javanica*	Phytohormonal signaling	Gene expression/functional analysis	downregulated/negative regulator of plant immune response	Activation of Pattern-triggered immunity leads to the production of reactive oxygen species and activation of signaling pathways involving MAPKS along the phytohormonal signaling	[21]
ERF109	*Arabidopsisa*/*M. incognita*	JA signaling	Gene expression using GUS staining and RT-qPCR	Upregulation/regulator of tissue regeneration	JA-induced ERF1109 triggers the expression of CYCD6;1 upstream of another ERF TF, to promote stem cell regeneration and activation	[110]
DEL1(E2F)	*Arabidopsis/M. incognita*	SA and lignin biosynthesis	Gene expression analysis	Downregulation/repression of defense-related genes	Modulates the expression of genes related to SA biosynthesis and lignification in response to RKN infection	[111]
GmEREBP1	Soybean*/Arabidopsis/H. glycines*	Hormone signaling	Expression analysis using qRT-PCR	Upregulation/activation of defense genes such as PR1	Acts as a transcriptional activator to promote the expression of defense-related genes	[112]
KNOX	Medicago/*M. incognita*	Hormone signaling	Gene expression analysis	Upregulation/regulator of polar auxin transport	KNOX expression is spatially restricted to the meristem, where phytohormone levels are regulated to influence the cellular responses for giant cell formation	[113]
LBD16,	*Arabidopsis/M. incognita*	Hormone signaling	Gene expression analysis	Upregulation/component of xylem pole pericycle (XPP) gene expression important for lateral root formation	Expression of LBD16 plays a role in the plant's response to stress by promoting cell proliferation in the pericycle	[114]
ARF8A/ARF8B	Tomato/*M. incognita*	Auxin signaling	Gene expression analysis	Upregulation/regulates downstream genes involved in the defense response	Reprogramming of gene expression is crucial to gall formation	[115]
ARF5	*Arabidopsis*/*M. Javanica*	Auxin signaling	Gene expression analysis using qRT-PCR	Upregulation/activator of developmental pathways	ARF5 has a role in regulating the plant's response to nematode infection by promoting developmental pathways that facilitate gall formation	[116]

Functional studies in *Arabidopsis* and tomato have identified 15 TF “hubs” co-regulated during infection, including MYB, WRKY, and ARF members [117, 118]. One such TF, DEL1, an atypical E2F member, suppresses SA accumulation and lignin biosynthesis, thereby promoting RKN infection. Mechanistically, DEL1 represses the expression of *EDS5*, a gene encoding a SA transporter involved in the isochorismate pathway, thereby reducing SA accumulation during *M. incognita* infection. In contrast, *del1* mutants show elevated SA levels, increased lignification, and resistance to RKN [111], emphasizing DEL TFs as modulators of defense. These mutants also show upregulated expression of lignin biosynthesis genes including PAL1, C4H, and CAD5, with corresponding intense lignin staining observed in galls a 5 dpi. This suggests that del1 plays a dual role in suppressing SA-mediated immunity and lignin deposition to favor nematode parasitism. Notably, *M*. *incognita* effector like Mi-ISC-1 also targets the isochorismate pathway to suppress SA defenses [119], further supporting the importance of this hormonal route in host-nematode interaction. Together, these findings highlight DEL1 as a critical modulator of the innate plant defense mechanism during gall formation.

The WRKY family also plays particularly diverse roles in plant growth and development, and stress responses [120, 121]. Despite the structural conservation of the WRKYGQK motif and zinc-binding domain, individual WRKYs show functional divergence [122, 123]. For example, *SlWRKY72a* and *SlWRKY72b* in tomatoes are upregulated during Mi-1-mediated resistance, while silencing both increases susceptibility [107], confirming their roles in plant basal defense. Interestingly, the AtWRKY72 orthologs in *Arabidopsis* confer similar basal resistance, but independent of SA signaling, suggesting a unique defense pathway.

In *H. schachti* infection, BHLH TFs such as BHLH25 and BHLH27 act synergistically to support syncytium development and later root morphology[124]. Overexpressing either enhances parasitism, while the double mutant shows little change, indicating partial redundancy but essential co-functionality. Notably, members of a transcription factor family can exhibit contrasting roles in plant defense. For instance, within the WRKY family, some members act as negative regulators of plant defense response [125], while others are positive regulators against nematode infections. This duality highlights the complexity of their regulatory functions.

As positive regulators, WRKY11 is strongly expressed 24 h after RKN infection, activating defense responses. Similarly, WRKY17 plays a partially redundant role alongside WRKY11, contributing to a PTI-oriented defense mechanism. However, upon nematode infection, both single and double mutant lines exhibited a similar number of galls, suggesting the lack of functional redundancy between WRKY11 and WRKY17. Furthermore, studies using the WRKY11 promoter::GUS *Arabidopsis* line revealed that WRKY11 expression is localized to the root elongation zone and tips as early as 24 h post-infection [105]. More so, *Arabidopsis* mutants for *wrky11*, *wrky17*, and the double mutant *wrky11*/*wrky17* demonstrated heightened susceptibility to both CN and RKN, with no significant difference in infection rate across the lines [105, 109]. These findings underscore the crucial role of WRK11 and WRK17 in plant defense, emphasizing their broad relevance across distinct nematode infection patterns.

The expression patterns of WRKY genes during CN infection further shed more light on their diverse and sometimes contrasting roles in host-parasite interaction. While some WRKY genes contribute to nematode development, others favor plant defense. Notably, WRKY6, WRKY11, WRKY17, and WRKY33 were found to be downregulated in the syncytia, a change that favors nematode activity, whereas WRKY40 and WRKY54 were upregulated in older syncytia only, suggesting a potential role in later feeding site developmental stages [109]. Interestingly, WRKY23, one of the upregulated genes, has been demonstrated to play a critical role in nematode development by inducing auxin-responsive genes that promote feeding site formation[126]. Understanding the complexity of their regulatory role is essential, especially how a gene contributes to syncytium formation and progression. Such observation points to a possible shift in gene function depending on the developmental stage of the syncytium, providing an intriguing area for further exploration.

Transcriptional profiling of infected tomatoes has revealed that WRKY genes are differentially expressed and often function as negative regulators during RKN infection [127]. For example, in investigating the functional roles of tomato WRKY genes *SIWRKY16* and *SIWRKY31* during nematode invasion, the promoter-GUS reporter gene analysis indicated that both genes are predominantly expressed during the early parasitic activities [21]. Moreover, the output of qRT-PCR of the tomato lines indicated that both genes reached maximum expression 15 days after inoculation, with *SIWRKY31* showing earlier induction but notable downregulation as the nematode matured. Functionally, overexpressing these genes enhanced the infection while suppressing the activity of defense-related genes, indicating their roles as negative regulators of the plant's defense against nematodes. In corroboration, [18] reported that several upregulated WRKY TF act as negative regulators of defense response to *M. javanica* infection.

Further analysis using a *SIWRKY45*promoter::GUS reporter line demonstrated intensive activation from 5 days post-inoculation through gall development and maturation stage, peaking around 28 dpi. In overexpression lines, defense marker genes associated with SA- and JA-mediated pathways, as well as cytokinin response factors (CRF1 and CRF6), were repressed, suggesting that *SIWRKY45* impairs the plant's ability to respond effectively to cytokinin. Interestingly, this coincides with findings that *H. schachtii* expresses an isopentenyltransferase gene, facilitating the synthesis of cytokinin required for virulence and feeding site maintenance [68, 128]. Consequently, the excessive cytokinin level driven by nematode infection may disrupt plant hormone signaling, further altering the plant's innate immune mechanism.

Notably, the evidence supports that certain WRKY members function as negative regulators of plant innate defense, aligning with the observed increase in nematode infection upon SIWRKY45 overexpression. This reinforces its role as a key mediator during PTI responses. In essence, manipulating *SIWRKY45* is crucial for the invading nematodes to disrupt the hormonal signaling, enabling their advancement within the host plant. Interestingly, SIWRKY45 has been identified as a major repressor of JA signaling through two key mechanisms. First, it directly interacts with SlJAZ proteins, members of the JA-ZIM domain (JAZ) family, which are established repressors of JA responses [129]. Under normal conditions, SlJAZ binds and inhibits SIWRKY45, preventing it from affecting downstream targets. However, upon JA-Ile accumulation, the SCF/COI1 complex mediates degradation of SlJAZ proteins, thereby releasing SlWRKY45. Once released, SlWRKY45 binds to the promoter of ALLENE OXIDE CYCLASE (AOC), a key enzyme in JA biosynthesis, leading to attenuation of SlAOC expression [129]. This causes a reduction in JA levels and a repressed defense response in tomato, ultimately facilitating nematode parasitism. These findings suggested that understanding these regulatory mechanisms could aid breeders in developing plants resistant to such manipulation by RKN.

Another group of TFs in nematode parasitism is the KNOX TFs, which exhibit significant upregulation in RKN feeding sites. Usually, KNOX TFs have roles in maintaining meristem functions and regulating lateral organ development in uninfected plants [113]. Remarkably, KNOX expression in nematode-infected plants is modulated by a sister TF, PHAN, demonstrating heightened expression in giant cells [130]. Additionally, the co-expression of genes encoding the early nodulation mitogen ENOD40 and the mitotic inhibitor ccs52 in giant cells [113] suggests overlapping regulatory pathways during parasitic influences and symbiotic nodulation. Taken together, the findings emphasize the shared molecular framework underlying host responses to parasitism and symbiosis, highlighting potential targets for enhancing plant resistance.

The ethylene-responsive element binding proteins (EREBPs) TFs are also strongly associated with plant defense responses, particularly through their interaction with promoter elements of pathogenesis-related (PR) genes [131]. One notable example is *GmEREBP1* from soybean, which is significantly downregulated during cyst-nematode parasitism [112]. Overexpression of *GmEREBP1* has been shown to activate various PR genes, thereby enhancing the ET- JA- and SA-mediated defense pathways. Interestingly, even though susceptible soybean cultivars exhibit reduced PR gene expression, transgenic lines overexpressing *GmEREBP1* show altered susceptibility to nematode infection [112]. GmTINY, a nuclear-localized AP2/ERF TF, plays a crucial role in soybean defense against SCN by binding to the ERELEE4 promoter elements within the SCN resistance locus Rhg1 [132]. Notably, GmTINY activates the expression of resistance genes such as GmAATRhg1 and GmSNAP18, which contribute to cell wall remodeling and hinder nematode development. Similarly, the AP2/ERF TF GmERF071, which harbors a functional domain associated with SCN resistance, confers enhanced resistance when overexpressed, highlighting its potential as a candidate gene for breeding SCN-resistant soybean cultivars [133]. Collectively, this highlights the role of *EREBP1* and the ERF TF as inducers of phytohormone-dependent defense pathways during nematode infection.

Similarly, the LATERAL ORGAN BOUNDARIES DOMAIN 16 (LBD16) TF promotes lateral root formation under the influence of the auxin pathway through AUXIN RESPONSE FACTORS (ARF) [114]. Specifically, *ARF7* activates the transcription of *LBD16*, *LBD18*, and *LBD29*, which are crucial for lateral root initiation by breaking the symmetry of the pioneer cells [134]. During RKN infection, the auxin-insensitive mutant tomato line shows significant reductions in infection rates [49, 135]. Remarkably, *LBD16* is activated by a nematode effector localized in the pericycle cells with a concomitant increase in auxin levels in the galls of marker lines, suggesting its critical role in cell reprogramming leading to the development of feeding sites [114]. This, taken together, indicates the intricate relationship between feeding site establishment, lateral root development, and auxin signaling during nematode infection.

Auxin signaling, a pivotal mechanism during feeding site development, is regulated by ARFs, which function as key transcription factors. For example, *ARF8A* and *ARF8B* effectively regulate auxin-inducible genes downstream in the nematode-infected plant [115]. Notably, transcriptome analysis reveals post-transcriptional regulation of ARFs during nematode infection in *Arabidopsis* and tomato, modulating genes such as SMALL AUXIN-UPREGULATED RNA (SAUR), with some being repressed or differentially expressed during the infection process [115, 136]. Likewise, another critical member, *ARF5*, is involved in gall formation by regulating the transcription of *GATA23* through interactions with other ARFs like *IAA28*. The gene is induced during early plant infection, interacting with other auxin-response factors such as IAA28. Hypomorphic mutants’ line arf5-2 shows reduced gall formation, further emphasizing its importance in developmental pathways leading to feeding site formation [116]. Together, these findings highlight that feeding site organogenesis relies on molecular regulators shared across various developmental pathways.

#### Nematode effectors manipulate Transcription processes to modulate plant cell development.

Nematode effectors are effective in various host manipulation mechanisms, including targeting transcriptional regulators that govern cell development and differentiation [137]. While much attention has been given to their defense-suppressive roles, increasing evidence highlights their capacity to reprogram host gene expression by modulating TFs and RNA processing elements.

**Fig. 2:**
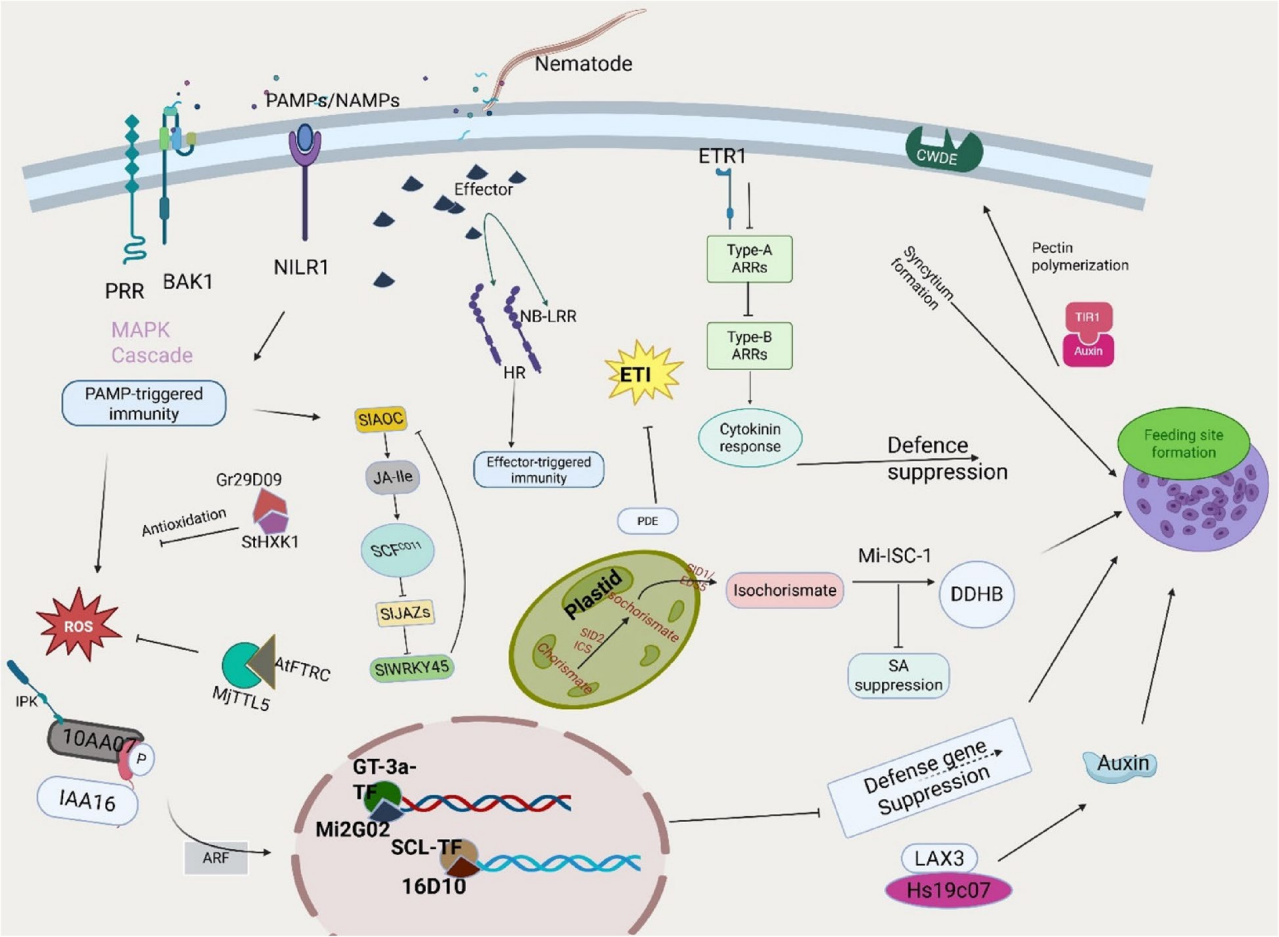
Molecular Dynamics of Nematode Feeding Site Formation. The formation of nematode feeding sites involves complex molecular reprogramming of host root cells, modulated by nematode effectors, plant immune signaling, and hormonal cross-talk. Pattern recognition receptors (PRRs) such as BAK1 detect pathogen/nematode-associated molecular patterns (PAMPs/NAMPs), activating MAPK cascades and initiating PAMP-triggered immunity. Effectors secreted by the nematodes subvert host defenses by targeting immune receptors (NB-LRRs), transcription factors, and hormonal pathways, suppressing defense responses, and enabling feeding site development. Key effectors include Mi2G02, which interacts with GT-3a to repress defense gene expression via inhibition of the 26S proteasome. 16D10 targets the SCARECROW-like transcription factors (SCL-TF) to promote root cell differentiation. 10A07 binds to IAA16 and interacts with IPK kinase to modulate nuclear translocation and the auxin pathway. Hs19C07 stimulates auxin through LAX3, leading to cell wall remodeling via CWDE activation. Additionally, Mi-ISC-1 suppresses salicylic acid (SA) accumulation by degrading isochorismate, a key SA precursor [119]. SlWRKY45 represses JA biosynthesis by interacting with JAZ proteins and downregulating AOC, reducing JA-mediated defenses [129]. Ethylene signaling (ETR1) modulates cytokinin response and syncytium formation through ARR factors. Together, these interactions reflect the nematode's capacity to manipulate ROS signaling, hormone transport, and transcriptional networks to establish and maintain specialized feeding sites, exploiting the developmental plasticity of host cells. NB-LRRs, nucleotide-binding leucine-rich repeat receptor Created in https://BioRe nder.com

Emerging studies have identified specific nematode effectors and their host targets during infection (Fig. 2). For instance, *M. incognita* effector Mi16D10 interacts with SCARECROW-like (SCL) TFs of the GRAS family to promote giant cell formation [16]. Overexpression of Mi16D10 in plants results in accelerated root growth, highlighting its function in reprogramming parasitized host plant cells [16]. Similarly, the effector Hs10A07 from *H*. *schachtii* interacts with *IAA16*, an auxin-responsive TF, altering auxin signaling pathways critical for NFS initiation [77].

Trihelix TFs, known for their role in development and stress responses, are also exploited. The *M. incognita* effector Mi2G02, for instance, interacts with the *A. thaliana* GT-3a, a member of the trihelix family, to enhance parasitism [15]. In confirmation of the dialogue, silencing GT-3a in the host led to attenuated feeding site development and low egg mass production. Functionally, GT-3a acts as a transcriptional inhibitor by binding to the TOZ and RAD23C promoters, thereby manipulating plant cell development in favor of the nematode [15]. This suppression is achieved through Mi2G02 inhibition of the downstream 26S proteasome-dependent pathways, demonstrating how nematodes manipulate host TFs to reprogram gene expression for their benefit.

The 30D08 effector protein plays a pivotal role in nematode manipulation of the host cellular process by targeting AtSMU2, a spliceosome component involved in pre-mRNA splicing and transcription regulation [102]. Although direct interactions between AtSMU2 and specific TFs have not yet been characterized in the context of nematode infection, SMU2 has been implicated in transcriptional regulation through its association with co-transcriptional splicing complexes and chromatin remodeling factors [138]. These suggestive links point to a broader regulatory role in gene expression during infection. For example, RDM16, another splicing factor, was found to be highly upregulated in SMU2-30D08 lines with a role in the DNA methylation pathway [139]. Moreover, functional studies support the importance of 30D08 in infection as silencing this effector via plant-derived RNAi significantly reduces nematode susceptibility [102]. Similarly, impaired nematode development in *smu2* mutant roots underscores the necessity of AtSMU2 for the infection process. Given 30D08's involvement in alternative splicing events, it will be interesting to examine other genes upregulated by the effector, as this will give additional insight into the mechanisms of host cell alteration in feeding sites.

Similarly, the effector MiEFF18 targets SmD1, a core spliceosomal protein, to alter alternate splicing during giant cell formation [140]. SmD1 regulates the processing of transcripts involved in cell cycle and cytoskeletal dynamics, suggesting that nematodes exploit splicing to fine-tune host development. Together, these effectors reveal a coordinated strategy by nematodes to hijack host transcriptional and post-transcriptional machinery. By targeting transcription factors, spliceosome components, and cytoskeletal regulators, nematodes reprogram plant cell differentiation and promote the formation of specialized feeding sites. Future studies should focus on mapping the interaction networks between effectors, TFs, and RNA processing factors to uncover key regulatory hubs for crop protection strategies.

While hormones and TFs are central regulatory elements during nematode infection, their expression and activity are also under epigenetic control, emphasizing the complexity of the interphase between defense response and parasitism.

#### Epigenetic regulation of transcription factors in nematode-infected cells

Beyond direct transcriptional control, epigenetic mechanisms, such as DNA methylation and chromatin remodeling, also play critical roles in reprogramming plant cells during nematode infection. These mechanisms induce specific changes in gene expression without altering the sequence but affect key processes such as cell cycle control, cell wall modification, and hormonal signaling during feeding site formation [97, 141]. For example, chromatin remodeling proteins, such as CHR19 and SNF4, are recruited to alter the accessibility of the transcription factor's binding sites, thereby modulating downstream gene expression [142, 143].

DNA methylation at the promoter regions of transcriptional factors plays a significant role in regulating gene activation or repression [144, 145]. Notably, hypomethylation events have been reported early during nematode infection, suggesting a broad, initial defense response in plants. this epigenetic relaxation may facilitate the activation of genes associated with stress and immunity. Conversely, de novo DNA methylation mediated by the RNA-directed DNA methylation (RdDM) pathway can silence gene expression by targeting specific loci, potentially favoring susceptibility.

In parallel, histone modification also contributes to transcriptional reprogramming. For instance, *H*. *schachti*i effector Hs32E03 interacts with histone deacetylase (HDAC) HDT1 and FK506 binding protein in the *Arabidopsis* cell nucleus, leading to altered acetylation patterns in rDNA regions, which promotes gene expression changes necessary for feeding site formation [146]. Since histone acetylation typically enhances transcription by loosening chromatin structure [147], nematode-induced deacetylation may repress certain gene sets while promoting others needed for parasitism. Furthermore, several nematode effectors are known to directly target the host nucleus to modulate chromatin status and reprogram gene expression [82, 140].

These rapid histone modifications are crucial for transcriptional reprogramming during nematode infection. Despite the progress with transcriptomic approaches, comparative analyses of epigenetic mechanisms combined with an understanding of effector-host interactions could provide deeper insights into cellular reprogramming and plasticity during nematode parasitism. Such knowledge has the potential to inform novel and targeted nematode management strategies.

So far, molecular studies based on transcriptomics approaches have enabled substantial progress in understanding the major transformation of root cells to create special feeding structures. However, comparative analyses of the mechanism involved, along with the effector and host-derived factors mediating the reprogramming and induced plasticity, could lead to novel nematode management strategies.

### Future directions and applications

The evidence linking cellular plasticity to nematode infection has firmly established the importance of transcription factors, hormonal signaling, and epigenetic reprogramming in feeding site formation. Advancing our understanding of plant cellular plasticity and the molecular interplay between plant defenses and nematode-induced reprogramming has established the roles of effector, host targets, and signaling pathways in nematode parasitism. These insights hold immense translational potential for developing innovative nematode management strategies.

Building on these insights, we explore some targeted approaches to mitigating the challenges facing the exploitation of molecular breeding for nematode management. Furthermore, we bring to the limelight the importance of advancing the understanding of epigenetic modulation and gene-silencing tools in shaping breeding strategies for nematode management.

#### Exploiting transcription factors for enhanced resistance

A deeper understanding of transcriptional factors that mediate cellular plasticity during nematode infection can directly inform resistance breeding. Specifically, identifying and characterizing TFs that are key to plant cell reprogramming during nematode infection is necessary. For example, TFs implicated in hormonal regulation and cell differentiation are promising targets. Through advanced gene-editing tools like CRISPR/Cas, it is possible to modify these TFs to avoid nematode-induced reprogramming while maintaining plant innate processes. By leveraging such precise molecular interventions, durable resistance against nematodes can be achieved.

#### Epigenetic modulation as a tool for resistance breeding

Studies have shown that epigenetic changes, such as DNA methylation and histone modifications, influence plant susceptibility and nematode resistance [97, 143]. Therefore, identifying specific epigenetic marks associated with resistance provides an opportunity to incorporate these traits into breeding programs. For instance, DNA methylation patterns in promoter regions of defense-related genes could be targeted to enhance the expression of such genes. Similarly, understanding the role of small RNAs and miRNAs in regulating gene expression during nematode infection can expand our toolkit for breeding crops with improved resilience.

Additionally, functional characterization of nematode effectors, their host targets, and how they modulate epigenetic mechanisms will shed more light on vulnerabilities that could be exploited for resistance. Moreover, it seems nematode effectors keep expanding as more are been discovered, so it is important to study how they are evolving and adapting to expanding host range, perhaps vulnerabilities in the nematode population could be identified, and/or emerging threats could be timely noted. This seems to be a worthy direction, especially now that assessing the full PPN effector repertoire is possible due to advances in gland-cell-specific transcriptomics [31]. More so, effector studies should target both the plant development- and physiology-altering groups, and those that suppress plant immunity, for a better grasp of effector functions.

#### Optimizing RNA interference (RNAi) technology

The application of RNA interference (RNAi) technology has been a handy gene-silencing tool used in targeting essential nematode genes for facilitating parasitism. However, the development of the technology for throughput functional genomics is faced with several challenges. Despite its hallmark in gene silencing and functional analysis, its application in plant-nematode interaction still encounters several technical and biological bottlenecks, such as delivery, differential efficiency, and stability of effect [148, 149]. The classical soaking of nematodes in dsRNA solutions is promising but lacks optimization and is yet to be fully adapted for high-throughput screening. Addressing these issues will be crucial for developing gene delivery systems and the optimized implementation of RNAi in functional genomics and nematode management.

In addition, there is potential to facilitate the modulation of plant hormone signaling or specific molecular pathways involved in defense and reprogramming. To address this, host-induced gene silencing (HIGS), a technique that targets essential nematode genes for infection [150], can be used to disrupt effector function and mitigate their ability to manipulate plant hormone pathways. Therefore, pursuing this strategy may open avenues for developing novel nematode control strategies. While these are strategies highlighting the potential for mitigating nematode parasitism, they display a complex plant-nematode interaction, which suggests the necessity of a thorough, multifaceted approach that integrates genetic and hormonal factors for effective and sustainable nematode management.

#### Emerging technique: single-cell RNA sequencing and proteomics

As discussed, nematode effectors reprogram plant development by targeting TFs, including SCLs, trihelix, and auxin-responsive proteins. However, bulk RNA-seq, which averages expression across tissues, may not resolve the heterogeneity of these TF responses within feeding sites like giant cells and syncytia. This limits our understanding of how individual cells contribute to effector-induced reprogramming. Moreover, functional redundancy among TFs often masks the effect of silencing a single gene [148], and bulk approaches cannot isolate immune or developmental responses in a specific cell profile. Single-cell RNA sequencing (scRNA-seq) overcomes these challenges by profiling gene expression at cellular resolution, revealing distinct cell populations, differential TF activity, and specialized responses to nematode infection.

Recent applications in helminth-infected CD4 + T cells [151] and arbuscular mycorrhizal symbiosis [152] highlight the power of this method in uncovering functional cell states during plant–microbe interaction. Applying scRNA-seq to nematode-infected roots could reveal how transcriptional plasticity varies across feeding site cells. However, the issue of potential mechanical damage during the enzymatic isolation of cells from the bunch has been raised [153]. Remarkably, snRNA-seq, which depends on nuclei isolation, has recently become a promising alternative to overcome the challenges of cell isolation. Altogether, snRNA-seq offers powerful tools to map effector-TF interactions, resolve transcriptional heterogeneity, and uncover reprogramming events central to nematode parasitism.

## Conclusions

The molecular mechanism underlying plant-nematode interactions represents a complex interplay between nematode-induced cellular reprogramming and the plant's defense responses. Sedentary endoparasitic nematodes utilize sophisticated strategies to manipulate plant cells into forming specialized feeding sites while evading immune responses. Remarkably, the process is fostered by the ability of plant cells to take different forms, under intricate regulatory networks involving transcription factors and hormonal signaling pathways.

Despite significant progress, several critical gaps remain. These include limited understanding of how specific TFs are modulated during different stages of infection, and how their interaction networks contribute to cells'trans differentiation into feeding structures. Notably, the mechanistic roles of TF cooperativity, epigenetic modulators, and targeted regulation, particularly the spatiotemporal modulation of epigenetic marks that direct transcriptional reprogramming during parasitism, are still not fully resolved. More so, understanding the role of long-distance signaling, including shoot-to-root, root microbiome interactions, during resistance or susceptibility mode is crucial.

Future research could target these regulatory mechanisms at single-cell resolution to uncover cell-specific responses. Emerging technologies such as single-cell RNA sequencing, epigenomics, and spatial transcriptomics will be crucial in addressing cellular heterogeneity within galls and syncytia. However, integrating these molecular insights into applied breeding strategies remains a critical challenge. Manipulating transcriptional regulators, including TF hubs like WRKY, MYB, and DEL1, or editing their regulatory circuits through CRISPR/Cas-based tools and RNAi, could offer effective breeding programs. Thus, while it is clear that RKN parasitism exploits cellular plasticity, we believe a deeper understanding of the underlying transcriptional reprogramming could offer a novel insight into molecular breeding strategies against nematode infections.

These efforts must be complemented by translational routes that bridge laboratory findings with crop improvement programs. Collaborative efforts between academic and industrial stakeholders are vital to realize these innovations in the field and address the global challenges posed by nematodes, and contribute to food security amidst increasing biotic stresses.

## Data Availability

Not applicable.

## Abbreviations

IPK: Inositol Polyphosphate Kinase
ARF: Auxin Response Factor
AUX: Auxin/Indole-3-Acetic Acid Protein
LAX3: Like-AUX1 3
TIR1: Transport Inhibitor Response 1
AFB: Auxin Signaling F-Box Protein
CWDE: cell wall degrading enzyme
IAA16: Auxin/Indole-3-Acetic Acid protein 16
ETR2: EIN3-Targeting protein
ERF: Ethylene Response Factor
EIN3: Ethylene Insensitive 3
ETI: effector-triggered immunity
ROS: reactive oxygen species
ICS/SID2: isochorismate synthase/Salicylic acid induction deficient 2
JA-Ile: jasmonoyl-isoleucine
SCF: Skp–Cullin–F-box complex
JAZ: jasmonate-ZIM-domain protein
BAK1: Brassinosteroid Insensitive 1-Associated Receptor Kinase 1
MAPK: mitogen-activated protein kinase
